# An Intricate Connection between Alternative Splicing and Phenotypic Plasticity in Development and Cancer

**DOI:** 10.3390/cells9010034

**Published:** 2019-12-21

**Authors:** Giuseppe Biamonti, Lucia Infantino, Daniela Gaglio, Angela Amato

**Affiliations:** 1 Institute of Molecular Genetics (IGM); National Research Council (CNR), 27100 Pavia, Italy; giuseppe.biamonti@igm.cnr.it (G.B.); lucia.infantino@igm.cnr.it (L.I.); 2 SYSBIO.IT, Centre of Systems Biology, University of Milano-Bicocca, 20126 Milano, Italy; daniela.gaglio@ibfm.cnr.it; 3 Institute for Molecular Bioimaging and Physiology (IBFM), National Research Council (CNR), 20090 Segrate (MI), Italy

**Keywords:** alternative splicing, cellular plasticity, tumor heterogeneity, cancer stem cells, EMT, cancer metabolism, neo-angiogenesis

## Abstract

During tumor progression, hypoxia, nutrient deprivation or changes in the extracellular environment (i.e., induced by anti-cancer drugs) elicit adaptive responses in cancer cells. Cellular plasticity increases the chance that tumor cells may survive in a challenging microenvironment, acquire new mechanisms of resistance to conventional drugs, and spread to distant sites. Re-activation of stem pathways appears as a significant cause of cellular plasticity because it promotes the acquisition of stem-like properties through a profound phenotypic reprogramming of cancer cells. In addition, it is a major contributor to tumor heterogeneity, depending on the coexistence of phenotypically distinct subpopulations in the same tumor bulk. Several cellular mechanisms may drive this fundamental change, in particular, high-throughput sequencing technologies revealed a key role for alternative splicing (AS). Effectively, AS is one of the most important pre-mRNA processes that increases the diversity of transcriptome and proteome in a tissue- and development-dependent manner. Moreover, defective AS has been associated with several human diseases. However, its role in cancer cell plasticity and tumor heterogeneity remains unclear. Therefore, unravelling the intricate relationship between AS and the maintenance of a stem-like phenotype may explain molecular mechanisms underlying cancer cell plasticity and improve cancer diagnosis and treatment.

## 1. Introduction

Tumorigenesis is a multistep process requiring the ability of cancer cells to survive in a challenging microenvironment. Indeed, during cancer progression, tumor cells undergo phenotypic changes due to a selective pressure established in the tumor microenvironment (TME) that depend on nutrient deprivation, hypoxia, and interactions with surrounding cells. A major contribution to the phenotypic plasticity of cancer cells comes from activation of embryonic mechanisms that are exploited to ensure survival and spread. For example, activation of the epithelial-to-mesenchymal transition (EMT) increases stem properties of cancer cells, enabling resistance to stress signals, evasion of apoptosis, and chemo-resistance. In addition, EMT promotes tumor spread by sustaining the migration and invasive behavior of cancer cells [1]. 

Similarly, re-activation of Wnt, Hedgehog, and Notch [2,3] signaling pathways or overexpression of transcription factors such as *OCT4*, *KLF4*, *SOX2*, c-*MYC*, and *NANOG* [4,5] in adult cells correlates with undifferentiated phenotypes, self-renewal capacity, quiescence, and resistance to several stress signals such as hypoxia and nutrient deprivation [6,7]. 

The molecular mechanisms that form the basis of this plasticity and their potential clinical implications remain to be fully explored, and this could represent a challenge for the improvement in the diagnosis and treatment of tumors.

The advent of deep sequencing technologies has shown that cancer cells originating from the same tissue exhibit different molecular profiles (intertumor heterogeneity). Surprisingly, this variability is also found within the same tumor bulk (intratumor heterogeneity). Effectively, subpopulations of cancer cells have been identified in several human tumors from hematological malignancies to carcinomas of different origins (i.e., breast, colon, pancreas, gastric, brain) [8,9,10,11]. Interestingly, these cancer cell subpopulations are endowed with stem-like properties and exhibit great adaptive behaviors. This high plasticity is an important cue for development of chemo-resistance mechanisms, evasion of immune surveillance, and spread to distant sites. Therefore, tumor heterogeneity is considered the main cause of therapy failure and cancer progression towards worse outcomes [12]. 

In this scenario, it is not surprising that defects in mechanisms controlling gene expression might have a pivotal role in cancer cell plasticity. 

In this issue we propose to explore the role of alternative splicing (AS) in phenotypic plasticity and its implication in human cancer. 

It is well known that AS regulates several biological processes such as proliferation, cell death, migration, and angiogenesis because it controls gene expression at the transcriptional level. Indeed, it increases the diversity of transcriptome and proteome in human cells, therefore, its deregulation may greatly contribute to tumor plasticity [13,14]. Effectively, AS plays a key role in the regulation of the balance between pluripotency and differentiation of human embryonic stem cells (hESCs) during embryogenesis and tissue differentiation. This consists in a proof-of-concept where defective AS machinery could sustain the acquisition of phenotypic plasticity in a pathological context [15].

This review highlights the contribution of AS to cellular plasticity in both physiological and pathological circumstances. 

First, we will explain the complexity of the AS network, owing to the involvement of cis-elements, spliceosome assembly, and regulation of a plethora of trans-elements with antagonistic functions that provide fine regulation of RNA maturation. Then, we will discuss the role of AS in regulation of the balance between pluripotency and differentiation during early embryogenesis and, at later stages, during tissue differentiation. Last, we will explore how aberrant AS affects stemness and phenotypic plasticity in human cancers, focusing on the role that AS has in biological processes such as EMT, adaptive responses to metabolic stress, and neo-angiogenesis.

In summary, we will show how defective AS mechanisms may support survival of cancer cells even in a challenging microenvironment. 

In-depth understanding of splicing regulation may provide new prognostic and predictive markers and suggest new anti-tumor strategies to address tumor heterogeneity and plasticity, such as the development of specific immune-stimulatory therapies.

## 2. The Intricate Molecular Mechanisms Controlling RNA Maturation

AS is a ubiquitous regulatory mechanism providing a fine-tuning of gene expression. Based on RNA-sequencing data, up to 94% of genes have intronic regions that are spliced during pre-mRNA maturation [16,17]. In addition, almost 80% of genes have alternative splicing variants that contribute to the establishment of physiological control of protein function at the transcriptional level.

Specifically, transcription of a multi-exon gene leads to the production of a nonmature pre-mRNA consisting of exons interspaced by intronic regions. Exclusion of intronic regions, through multiple splicing events, generates a mature mRNA whose sequence is read and translated into a functional protein.

**Figure 1:**
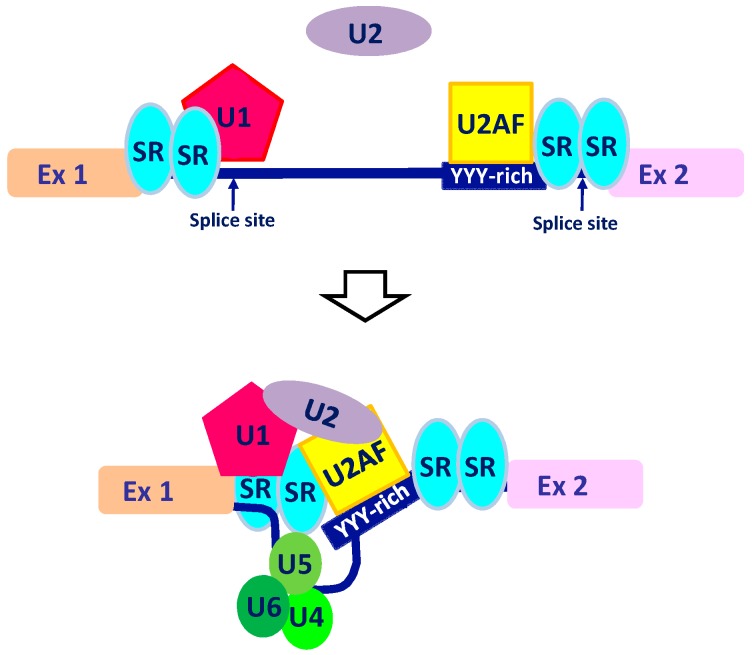
Assembly of spliceosome complex. Alternative splicing (AS) of a pre-mRNA follows different steps: U1 ribo-nucleoprotein binds to the 5′splice site (5′ss) while U2AF binds to the 3′splice site (3′ss) and the poly-pyrimidine tract (YYY-rich). In a second step, RNA-binding proteins (RBPs) (in the figure, serine/arginine-rich SR proteins) recognize and bind to the exon-splicing enhancer (ESE) sequence. The interaction between U1 ribo-nucleoprotein and U2AF (mediated by U2) promotes a conformational change of the RNA molecule favoring the binding of the tri-snRNP complex (U4–U5–U6 ribo-nucleoproteins).

As a multiprotein complex, the spliceosome recognizes splice sites at intron–exon junctions (5′ss and 3′ss) allowing RNA binding and maturation [18]. Specific RNA-binding proteins (RBPs), often characterized as U2AF and its antagonist PTB, cooperate with spliceosomes during the splice-site recognition (Figure 1).

Considered masters of AS, RBPs are responsible for recognition and binding of cis-elements at exon–intron junctions. Consequently, they enhance or repress intron or exon splicing through binding of consensus sequences: intron- or exon-splicing enhancers (respectively, ISE and ESE) and intron- or exon-splicing silencers (respectively, ISS and ESS). It follows that variations in RBP expression levels deeply influence gene expression [19].

Two main groups of ubiquitous RBPs have been characterized in detail: hnRNPs (heterogeneous nuclear ribo-nucleoproteins) and SRs (serine/arginine-rich) families. SR proteins are considered splicing activators. The C-terminus serine-rich domain (RS domain) regulates protein–protein interactions, whereas the RNA-binding domain (RRM domain) allows pre-mRNA binding. Conversely, hnRNPs have an inhibitory activity. In addition to ubiquitous SRs and hnRNPs, several tissue-specific splicing factors have been identified [20,21,22,23,24]. So far, the best characterized AS regulators are NOVA1/2, PTBP2, SRRM4, and members of the RBFOX, MBNL, CELF, TIA, ESRPs, and STAR families.

**Figure 2:**
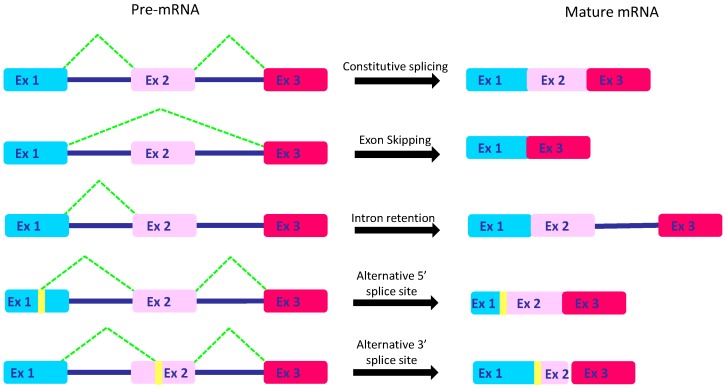
Mechanisms of AS. A three-exon pre-mRNA with two intronic regions (in the example) could be spliced into a mature mRNA through recognition of constitutive splice sites and removal of intronic regions. Otherwise, exon 2 is lost through exon skipping or intron 2 may be included in the mature RNA through intron retention. Alternatively, pieces of exon 1 or exon 2 may be lost when the spliceosome recognizes, respectively, alternative 5′ss or 3′ss. Altogether these mechanisms affect mRNA sequence and, consequently, the expression, localization, and function of the protein.

Alternative isoforms could originate from distinct mechanisms such as exon-skipping or intron-retention promoting, respectively, exon exclusion or intron inclusion into the mature mRNA. Moreover, sometimes spliceosome might ignore the constitutive splice site, preferring alternative ones located in the exon or intron sequences. Therefore, a piece of exon could be lost or intronic regions could be included [25] (Figure 2).

Together, these mechanisms profoundly affect gene expression at the post-transcriptional level because they regulate mRNA stability, localization, and translation. Hence, it is not surprising that AS is required for proper gene expression/repression during embryo development and tissue differentiation [26]. In addition, it contributes to tissue homeostasis in adult organisms. Accordingly, AS could be modulated by several intracellular and extracellular stimuli, regulating the balance between antagonistic splicing factors. 

This suggests that AS is a flexible and dynamic mechanism that may greatly contribute to the adaptive capacities of cells, regulating phenotypic plasticity [27]. Importantly, there is a strong correlation between AS and epigenetic modifications. Indeed, DNA methylation, chromatin remodeling, and nucleosome occupancy influence Pol II slow down or acceleration and affect the recognition of splicing sites or RBPs recruitment [28,29,30].

As a consequence, mutations in cis- or trans-elements and epigenetic changes affecting AS predispose cells to several human diseases. 

## 3. AS in Development

### 3.1. A Finely Regulated AS Network Controls Human Embryonic Stem Cells (hESCs) Pluripotency during Embryogenesis

During embryogenesis, a multicellular organism is generated from a single cell. The ability of each single cell to proceed along a lineage-specific differentiation path is driven by pervasive gene expression reprogramming [26,31] in which AS has a pivotal role [17].

Effectively, established AS events in specific genes constrain the pluripotency of human embryonic stem cells (hESCs). In order to identify the sequence of AS events that occurs during differentiation of hESCs, a previous study compared RNA-seq data of four cell types originating from hESCs (trophoblast-like cells, mesoendoderm cells, mesenchymal stem cells, and neural progenitors). This study revealed that AS events increase as development proceeds, in addition, AS isoforms are lineage-dependent [32]. 

Recently, several splice variants have been shown to play key roles in lineage commitment and differentiation [33,34,35]. For example, the analysis of receptors and secreted factors in hESCs through a DNA microarray found a correlation between high amounts of the fibroblast growth factor 4 (FGF4) and pluripotency. Indeed, *FGF4* is a target of the heterodimeric transcription complex SOX2/OCT3 [36,37], however, its expression is down-regulated at the transcriptional level during differentiation. AS of FGF4 produces FGF4si, coding for the N-terminal half of FGF4. This isoform is an antagonist of FGF4, opposing FGF4-induced ERK1/2 phosphorylation [38].

Similarly, Sall4 is a transcription factor that is essential for the pluripotency of hESCs. Two different isoforms, Sall4a and Sall4b, create homodimers and heterodimers, interacting with Nanog and having overlapping, but not identical, binding sites. The relative abundance of the two isoforms influences the interacting partners and the cell fate: Sall4b maintains the pluripotent state, whereas Sall4a promotes a switch towards differentiation [39].

The activity of other transcription factors is controlled by AS [33,38,39]. In some cases, the corresponding gene encodes for different splicing isoforms with opposite functions. For example, *OCT4A* and *OCT4B1* are primarily expressed in ESCs, whereas *OCT4B* is mainly expressed in differentiated cells [40]. Similarly to OCT4, different isoforms of FOXP1 are produced by AS in hESCs and act as a switch between pluripotency and differentiation [40,41]. 

Sometimes, stem-related transcription factors are regulated by inclusion of mutually exclusive exons. For example, hnRNP H1 and hnRNP F control the inclusion of the E12 or E47 exons of TCF3. Interestingly, the inclusion of the exon E47 is associated to cell differentiation, whereas E12 inclusion contributes to the maintenance of pluripotency [42].

Conversely, there are also some examples of transcription factors controlling AS. Indeed, SOX2 regulates the expression of *SRSF2*, an SR-splicing factor that plays a critical role in both constitutive and alternative pre-mRNA splicing [43]. In addition, it exhibits RNA-binding capability in vitro, likely for direct modulation of AS, as suggested by previous studies [44]. 

### 3.2. AS Isoforms Control Cell-Lineage Differentiation during Organogenesis

Recently, the high-throughput sequencing technologies combined with studies in animal models have made it possible to expand our knowledge of the physiological impact of AS networks during tissue differentiation. [17].

For example, differentiation of the brain and the skeletal muscle needs a deep reprogramming of gene expression that is tightly regulated by changes in RBPs [45]. For example, PTBP2 transcript encodes a protein that plays a critical role in neural differentiation and tissue maintenance. Maturation of its mRNA is tightly regulated by polypyrimidine tract-binding protein 1 (PTBP1) and serine/arginine repetitive matrix protein 4 (SRRM4), two RBPs differentially expressed during neurogenesis.

High levels of PTBP1 in proliferating neural stem cells maintain pluripotency because they promote exon 10 skipping of PTBP2 and the formation of a transcript with a premature termination codon. As cells exit the cell cycle to differentiate into neurons, PTBP1 is down-regulated. The simultaneous up-regulation of SRRM4 promotes exon 10 inclusion and accumulation of PTBP2 protein, triggering differentiation of neural stem cells [45].

Studies on mutant mice demonstrated that PTBP1 loss-of-function during development causes precocious neurogenesis and depletion of the neural stem cell pool. Moreover, *SRRM4* depletion inhibits mice neurogenesis of upper-layer neurons and causes the accumulation of progenitors or lower-layer neurons. This results in abnormal cortical lamination, whereas the loss of PTBP2 may alter neural stem cell localization and proliferation [45].

During neurogenesis, AS mechanisms also control neural migration and lamination through regulation of the Reelin signaling pathway. In particular, the AS factor NOVA2 (neuro-oncological ventral antigen 2) boosts the neural migration because it triggers AS of DAD1 (Disabled 1), a component of the Reelin signaling pathway. *NOVA2*^−/−^ mice, showing impaired AS of DAD1, suffered notable neuronal migration defects [45,46].

In addition, AS controls the generation of a neural network during synaptogenesis. Formation of functional synapses is a fundamental process for establishing neural circuits. Synaptic specificity is achieved by trans-synaptic adhesion between pre- and postsynaptic neurons. Importantly, the splicing factor SLM2 (also known as KHDRBS3) is responsible for AS of pre-synaptic neurexin (NRXN). The NRXN variants control the targeting of the postsynaptic partners [47], which are involved in plasticity of the neural network. Defective NRXN AS in Slm2^−/−^ mice dramatically impaired synaptic plasticity [48]. 

AS mechanisms have been extensively studied in differentiation of striated muscle (both cardiac and skeletal muscle). Previous genome-wide analysis showed that AS regulates the transition from embryonic stem cells to cardiac precursors up to postnatal heart development. 

Many physiological and morphological changes have been linked to AS events. The RBM20 protein is responsible for modulating the splicing of Titin in cardiomyocytes, a giant protein acting as a molecular spring [49]. The Titin AS is tissue-specific and contributes to the regulation of mechanical properties of the heart by producing two different isoforms: N2BA and N2B. The N2BA protein is primarily expressed in newborns; in contrast, the N2B protein is more abundant in adults. The region of Titin mRNA susceptible to AS controls the elasticity of the molecule, therefore, the ratio between the two isoforms establishes the cardiomyocyte passive tension as well as determines the sarcomere length and the myocardium wall stiffness [49]. 

Taken together, the above findings highlight the impact of the AS networks on cellular plasticity in physiological contexts.

## 4. Contribution of AS to Tumorigenesis

### 4.1. Defective AS in Human Cancers

**Figure 3:**
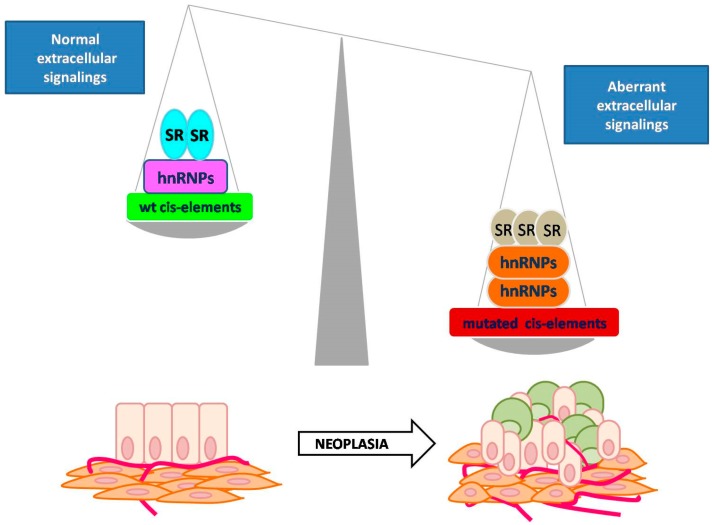
Connection between defective AS and tumor heterogeneity. Hypothetical mechanism explaining the connection between defective AS and tumor heterogeneity. AS has been shown as a mechanism regulating cell-lineage differentiation during embryogenesis. In adult tissues (on the left), the balance between antagonistic splicing factors (i.e., heterogeneous nuclear ribo-nucleoproteins) (hnRNPs) and SRs) contributes to the maintenance of cell differentiation. Cell adhesions and a well-defined epithelial shape (bottom left) characterize epithelial cells (light pink). In a physiological context, they receive oxygen and nutrients by blood vessels (red) and interact with surrounding stromal cells (orange). In a pathological context, aberrant extracellular signals or stochastic mutations dramatically affect the balance in antagonistic splicing factors (on the right) leading to tumor heterogeneity. Differentiated (light pink) and stem-like (orange) cancer cells coexist in the same tumor bulk. Their interaction with surrounding stromal cells may sustain neo-angiogenesis and activate invasive programs at later stages (bottom right).

The role of AS in development suggests that it could be an important player in controlling cancer cell plasticity and tumor heterogeneity. Indeed, if specific isoforms are necessary to maintain cell differentiation in adult tissues [50], then the perturbation of the AS network in cancer cells may lead to re-activation of stem pathways affecting cancer cell plasticity. It follows that defective AS could be a trigger for tumor relapse and metastatic spread, dramatically affecting patient outcome (Figure 3).

Interestingly, genetic variants affecting the RNA maturation process are as abundant as those affecting gene expression. This means that, likely, both mechanisms give an equal contribution to phenotypic diversity [51].

Mutations that disrupt cis-acting elements within individual genes or affect trans-acting components of the RNA-processing machinery have been found in several human diseases [52,53]. Some of these alterations have been previously reported [54,55].

The most common form of splicing defects is mutation in cis-elements such as splice sites, intron–exon junctions or enhancer/silencer sequences. For example, a mutation of the splice site in hSNF5, an ATP-dependent chromatin regulator, causes deletion of exon 7 and predisposes the person to pediatric brain cancer [56]. In colon cancer, a mutation in the splice site at the intron–exon junction leads to exon 4 skipping of APC (Adenomatous polyposis coli) and has been associated to liver metastasis [57]. Some splice site mutations could have a more detrimental impact on mRNA maturation. For example, mutated splice sites in MLH1 pre-mRNA lead to a double exon skipping, associated to hereditary non-polyposis colorectal cancer (HNPCC) [58].

In addition, it has been shown that mutations affecting spliceosome assembly and function also have pathological consequences. Indeed, mutations of PRPF6, an U5 ribo-nucleoprotein, have been found to be overexpressed in colon cancer and associated to uncontrolled proliferation [59]. Similarly, mutations in U2AF1 and U2AF2 disturb hematopoiesis because they severely impair splice-site recognition and spliceosome positioning. These mutations are drivers of several hematological malignancies because they affect mRNA maturation of multiple genes [60,61]. Similarly, SRSF2 mutations have a profound impact on the AS profiles of numerous genes, halting hematopoietic cell differentiation [62].

Interestingly, previous studies suggested that the imbalance between alternatively spliced isoforms could be associated to cancer onset and progression. For example, the full-length androgen receptor (AR) mRNA encodes for a protein that binds to androgenic hormones and activates transcription of responsive genes. Exon 3 exclusion determines the loss of a zinc-finger motif in the protein, affecting DNA binding and transcription function. In a physiological context, the two isoforms contribute to hormonal regulation of gene expression. However, the switch to the truncated isoform has been detected both in breast cancer cells and tissues, suggesting that the imbalance between alternatively spliced isoforms could be a trigger in breast oncogenesis [63].

Similarly, exon 7 exclusion in estrogen receptor (ER) pre-mRNA generates a protein lacking an estrogen-binding domain, affecting its ability to properly activate upon hormonal stimulation. Importantly, the accumulation of the truncated ER protein has been associated with progression from G1 to G3 stage in endometrial cancer and is correlated with endocrine resistance in breast cancer patients [63,64].

Taken together, the above findings are few but are significant examples showing the correlation between AS defects and cancer (for a thorough report, readers are referred to [14,54]).

A recent study further highlights the contribution of these alterations to cancer onset and progression. In order to establish tumor profiles based on analyses of AS splicing events and on expression of specific RNA isoforms, researchers created a comprehensive workflow to integrate analyses of RNA and whole-exome sequencing data from 8705 human cancer donors, including 670 matched normal samples, spanning a range of 32 cancer types [65]. They found a larger amount of AS events in tumor samples than in normal ones. They also identified the existence of tumor-specific splicing patterns linking AS to tumor heterogeneity. In particular, cancers that are commonly ascribed with similar characteristics, clustered closely together. Whereas, different breast cancer subtypes could be distinguished based on exon skipping events.

### 4.2. AS-Mediated Phenotypic Switch in Epithelial–Mesenchymal Transition

Epithelial–mesenchymal transition (EMT) is a reversible specialized process that occurs during the normal embryonic development, which is pivotal for cell differentiation and tissue patterning. Following the earliest stages of embryogenesis, the embryo implantation and formation of the placenta are both associated with EMT. In particular, the cells of tropho-ectoderm, which are polarized epithelial cells, detach from the basal membrane, acquiring a mesenchymal phenotype with high invasive capabilities and migratory activity. These modifications are required in order to facilitate invasion of the endometrium and the proper anchoring of the placenta, which supports embryo development [66,67,68,69]. This is only the first of many events in which EMT occurs during embryonic development (for more details, readers are referred to [70]).

This program also occurs during wound healing and is exploited by cancer cells to drive tumor progression and metastatic spread [71]. The following common traits define EMT activation: E-cadherin loss and nuclear translocation of beta-catenin and activation of transcription factors such as TWIST, SNAIL, and SLUG. The consequence is a phenotypic switch of cancer cells into a mesenchymal-like phenotype as shown by increased expression of mesenchymal markers, N-cadherin, and vimentin. Moreover, a profound reprogramming of gene expression, following activation of EMT-specific transcription factors, enables detachment from the basal membrane, degradation of the ECM (extracellular matrix), and invasion of distant organs [72]. 

Clearly, EMT increases phenotypic plasticity through re-activation of stem pathways [1]. Hence, EMT could be considered a trigger of tumor heterogeneity, generating subpopulations of stem-like cancer cells that exacerbate tumor aggressiveness. Indeed, EMT is frequently associated to metastatic spread and resistance of cancer cells to anti-cancer treatment and to apoptosis.

Interestingly, EMT robustly influences post-transcriptional processes of mRNA maturation [73]. For example, EMT regulates the splicing factor ESRP, promoting exon 4 skipping of TCF4, a member of the Wnt signaling that is activated by nuclear localization of beta-catenin. Isoforms that include exon 4 show reduced transactivation activity; conversely, exon 4 exclusion further enhances Wnt signaling during EMT. Indeed, mesenchymal cells predominantly show the truncated isoform [74].

Another target of AS is NUMB, which plays an essential function in the maintenance of cell polarity and cell–cell adhesion through E-cadherin binding. This interaction is likely to be promoted by a 33-nt epithelial-specific exon in NUMB. In cancer, EMT promotes NUMB exon skipping, supporting acquisition of invasive properties [75]. Interestingly, NUMB is also involved in asymmetrical division of stem-like cancer cells, therefore, aberrant AS may also affect the balance between stem-like and non-stem cancer cells [76].

The splicing factor ESRP also regulates the relative abundance of CD44 isoforms: CD44v variants are commonly expressed in epithelial cells, while CD44s are mainly found in hematopoietic and mesenchymal cells. The balance between the expression of these two isoforms appears deregulated during tumor invasion and metastasis, through EMT-mediated down-regulation of ESRP1 [77]. Indeed, in a Twist-inducible EMT system, overexpression of ESRP1 prevented the switch of CD44 into CD44s isoform and blocked EMT [78]. Importantly, the accumulation of CD44s isoform is significantly associated to recurrent high-grade breast cancer and increased stem-cell like subpopulation in breast carcinomas [78,79].

AS events during EMT also concern surface receptors. SRSF1, a splicing factor belonging to the SR protein family, stimulates skipping of exon 11 and production of ΔRON, considered a trigger of EMT [80]. Similarly, hnRNPA2/B1, a splicing factor that is altered in several cancers [81], also promotes exon 11 skipping. Conversely, ΔRON is inhibited by hnRNPA1. It follows that multiple splicing factors control a switch in EMT activation; moreover, an additional layer of complexity is due to the fact that SRSF1 itself is regulated through AS events by Sam68 [82]. Also of note, several human cancers express higher amounts of Sam68 and its overexpression has been linked to neoplastic transformation and tumor progression [83,84].

Interestingly, a mutually exclusive splicing of exon 3 of FGFR2 contributes to regulation of FGF signaling. Both mutually exclusive exons affect the extracellular domain of the receptor, therefore, they account for the different ligand-binding specificities of the two FGFR2 isoforms. In particular, exon 3b inclusion is predominant in epithelial cells, whereas a mesenchymal isoform is characterized by inclusion of exon 3c. Specifically, this AS event makes mesenchymal FGFR2 able to bind to the FGF of epithelial origin and vice-versa, ensuring proper crosstalk between epithelial and mesenchymal cells during embryonic development [85]. 

The latter evidence clearly shows how deregulated AS events might also affect cancer cell communication in the tumor microenvironment (TME). Importantly, TME is considered a great contributor to tumor heterogeneity and acquisition of phenotypic plasticity since it could support tumor growth and adaptation of cancer cells to metabolic stress and hypoxia [86]. 

Furthermore, it has been reported that EMT could be regulated by non-coding RNAs through modulation of AS events. For example, FGFR3 has been linked to tumor progression in many types of cancer and associated to EMT activation. FGFR3 AS is regulated by a small nucleolar RNA, snoRNA HBII-180C [87].

Recently, certain lncRNAs have been shown to play a crucial role in the regulation of AS in response to several stimuli or in some pathological circumstances. Frequently, they act as regulators of splicing factors, as demonstrated by MALAT1. This lncRNA is required for proper localization of SRSF1 to nuclear speckles. This event modulates the concentration of free SRSF1 within the cells, leading to significant changes in AS patterns of a subset of transcripts [88,89]. In addition, in ovarian cancer, MALAT1 represses the splicing factor RBFOX2, controlling pro-apoptotic tumor suppressor gene KIF1B [90]. Its pro-metastatic role is confirmed by the observation that MALAT-1 levels are significantly increased in primary tumors that subsequently metastasize.

In summary, AS emerges as an important determinant of cellular plasticity and the invasive behavior of cancer cells, contributing to worse outcomes in cancer patients.

### 4.3. AS: An Important Player during Metabolic Stress and Neo-Angiogenesis

During tumorigenesis, cancer cells experience hypoxia and nutrient deprivation due to the growth of the tumor bulk. In addition, the tumor microenvironment is susceptible to frequent changes induced by anticancer drugs. Phenotypic plasticity elicits an adaptive response to ensure survival and spread of tumor cells [8], defining a poor outcome in cancer patients.

Importantly, recent studies showed that tumor plasticity gives cancer cells the ability to establish new mechanisms for competitive glucose uptake [91,92]. In this scenario, it is not surprising that aberrant AS could emerge as a pivotal mechanism in the influence of the cellular response to metabolic stress. 

Recent studies suggest the existence of a connection between glucose metabolism and splicing programs. AS modulates the ratio between M1 and M2 isoforms of pyruvate kinase, in this way, determining the choice between aerobic glycolysis and mitochondrial oxidative phosphorylation (OXPHOS). In turn, metabolites of the Krebs cycle impact splicing programs at different levels by modulating the activity of 2-oxoglutarate-dependent dioxygenases (2-OGDDs) [93]. In addition, increased levels of NADH induce the activation of the transcriptional corepressor CtBP involved in cell adhesion, metastatic invasion, drug-sensitivity, and RNA metabolism [94].

AS emerges also as a key regulator of the target of rapamycin complex 1 (mTORC1). It is the better characterized of two mTOR-containing multiprotein complexes (the other being mTORC2) involved in the regulation of several anabolic processes [95] such as nucleotide, protein, and lipid synthesis [96]. The mTORC1-mediated protein synthesis is induced by activation of S6K [97], which in turn is under the post-transcriptional control of SRSF1. Basically, AS of S6K generates two different isoforms with opposing activities: The longer one inhibits mTORC1, impairing cell growth and transformation. The shorter isoform induces mTORC1 activity, resulting in increased oncogenic properties. Accordingly, SRSF1 overexpression promotes accumulation of the shorter one [98]. 

Many tumor cells exhibit increased rates of glucose uptake but reduced rates of OXPHOS. For a long time, this Warburg effect has been considered an inefficient means of ATP production, likely owing to mitochondrial defects. In contrast, recent studies showed that tumor cells choose different metabolic paths in a context-dependent manner [99].

Changes in AS events could make cancer cells able to survive stress signals because they might elicit metabolic rewiring during tumor progression. For instance, some genes encoding for glycolytic enzymes are susceptible to AS. Pyruvate kinase (PK) catalyzes the last step in glycolysis: conversion of phospho-enolpyruvate (PEP) to pyruvate [100]. Two different genes encode for PK: *PKLR* and *PKM*, each producing two different variants [101]. While *PKLR* is mainly expressed in liver and hematopoietic cells, the majority of tissues express *PKM*, which encodes PKM1 and PKM2 isoforms. Their difference consists in mutually exclusive exon 9 and exon 10, the first one included in PKM1, the second one included in PKM2. While PKM2 still retains cell-signal-dependent activation, PKM1 is constitutively activated. This reflects in accumulation of glycolytic intermediates and a boost in anabolic synthesis [102].

*PKM2* up-regulation is common to several cancers and contributes to a switch to glycolytic metabolism. This metabolic rewiring is driven by different signals from TME. For example, in an EGFR-activated cellular model, PKM2 could translocate into nucleus functioning as a beta-catenin co-activator. The PKM2/beta-catenin complex promotes transcription of c-Myc target genes such as *GLUT-1* (glucose transporter), *LDHA* (Lactate dehydrogenase A), and *PTPB1*. Effectively, nuclear translocation of PKM2 has been found in human glioblastoma. Conversely, the expression of a PKM2 mutant lacking a nuclear localization signal inhibited the EGFR-promoted Warburg effect and brain tumor development in mice [103].

It is worth noting that the positive loop between PKM2 AS and PKM2-mediated PTBP1 expression could be an important regulator of chemo-resistance in pancreatic ductal adenocarcinomas [104]. 

These findings represent an excerpt of the intricate connection between AS and phenotypic plasticity (for a more complete picture, readers are referred to [105]) and clearly show how AS variants could elicit an adaptive response to metabolic stress during tumorigenesis.

Another aspect of tumor metabolism affected by AS events is the response to hypoxia. Cancer cells of the inner bulk experience oxygen deprivation during tumor progression. Hypoxia is a common characteristic of many solid tumors and is finely regulated by a crosstalk between the tumor and surrounding cells. 

The hypoxic microenvironment stabilizes hypoxia-inducible transcription Factor 1α (HIF1α), promoting tumor cell survival. A recent study showed that breast cancer cells underwent extensive changes in AS if cultured in hypoxic conditions. Specifically, RNAseq data showed predominant intron retention events (among others, targets of AS included LDHA, TNFSF13, and ARHGAP4 for intron retention, MARCH7, PCBP2, and LRCH3 for exon skipping, and VGLL4, AHNAK, and NFE2L1 that are subjected to alternative first exon usage) [106]. A similar study also confirmed the occurrence of specific AS events during hypoxia in a cellular model of hepatoma [107].

Importantly, AS variants induced under hypoxia might potentially elicit different adaptive responses of cancer cells to this stress, for example, some of them could drive a metabolic switch to a glycolytic path while others could activate migratory activity supporting the escape from the primary tumor. Effectively, the expression of hypoxia markers in breast cancers, such as *HIF1α* and *CA9*, correlates with a more aggressive disease and poor prognosis [108,109].

Cancer cells acquire several mechanisms to face hypoxia, for example, they may drive the development of a new network of blood vessels, a process named neo-angiogenesis. 

Briefly, neo-angiogenesis sustains tumor growth and increases the chance of tumor spread. This process is triggered by paracrine signals [110] that stabilize HIF1α, the main activator of the angiogenic cascade. HIF1α accumulates in the cytoplasm and then translocates to the nucleus, promoting the transcription of genes with angiogenic potential such as *VEGF*, *ANGPT1*, and *TIE2*, platelet-derived growth factor (*PDGF*), *PDGFR* receptor, fibroblast growth factor (*FGF*), *FGFR* receptor, *NOTCH1*, and its ligand *DLL4* [111].

Many of these genes are regulated by AS mechanisms, as previously described in this review. In particular, the AS of VEGF ligands generates multiple variants with opposite functions, the most studied are VEGF-A isoforms. 

VEGF-A is spliced into VEGF-A_165_b in normal epithelial cells of the eye [112] and the kidney [113] whereas in cancer, the highly angiogenic VEGF-A_165_ isoform is predominant [114,115,116,117]. Interestingly, in colon cancer cell lines, there is a progressive switch from VEGF_165_b to VEGF_165_ expression during the progression from adenoma to carcinoma [117]. 

It is worth noting that SR proteins have been shown to regulate AS of VEGFA, specifically SRSF1 inhibition prevents in vitro and in vivo angiogenesis and tumor growth [118]. In addition, the AS factor U2AF65 and its partner JMJD6 are regulators of VEGFR1 [114].

Another important player in neo-angiogenesis is the splicing factor NOVA2, previously known as neural-specific splicing factor (see Section 3.2. “AS Isoforms Control Cell-Lineage Differentiation during Organogenesis” in this review). Recently, this factor has been shown to control the formation of vascular lumen. In particular, NOVA2 controls the AS of Par genes that deregulate endothelial cell polarity. Interestingly, *NOVA2* knock-down in vivo severely affected angiogenesis during development of zebrafish [24].

In summary, vascular homeostasis is regulated by a large number of pro- and anti-angiogenic factors that controls differentiation of progenitor endothelial cells. The above reported evidence provides proof of the involvement of AS mechanisms in controlling the differentiation capabilities of endothelial progenitor cells, therefore highlighting how the deregulated AS network could be a trigger of neo-angiogenesis in cancer. 

## 5. Conclusions

The advent of deep sequencing technologies has provided insight into the genetic complexity of malignant tumors. There is increasing evidence that, during tumor progression, cell subpopulations with distinct genomic alterations arise within the same tumor bulk, a phenomenon termed intratumor heterogeneity. This finding likely explains the failure of the conventional therapies that are not effective in contrasting tumor relapse and metastatic spread.

Phenotypic plasticity in cancer cell subpopulations seems to drive their survival in a challenging microenvironment because it elicits adaptive mechanisms to hypoxia, nutrient deprivation, or changes in the extracellular environment. Therefore, tumor heterogeneity is considered the main cause of cancer progression towards worse outcomes [12]. 

The re-activation of embryonic pathways has been suggested as a pivotal event, dramatically affecting tumor plasticity, since it increases the amount of poorly differentiated cancer cells endowed with stem-like properties. Compared to the majority of cancer cells of the tumor bulk, stem-like cancer cells easily acquire new mechanisms to escape immune-surveillance, become resistant to conventional drugs, and spread to distant sites.

Several factors may contribute to this gene expression reprogramming in human tumors, in this review we explored the role of alternative splicing (AS) and its implication in tumor progression.

AS is a flexible and dynamic mechanism accounting for post-transcriptional regulation of 94% of human genes. In particular, 80% of genes encode alternative splicing variants, contributing to the physiological regulation of protein function [16,17]. An intricate network of AS events constrains pluripotency of hESCs during embryo development. Moreover, a plethora of both AS splicing factors and alternatively spliced isoforms contribute to differentiation and maintenance of cell identity in the adult tissues. Accordingly, some studies have shown that AS events increase as development proceeds [32] 

This evidence clearly shows that AS mechanisms control phenotypic plasticity of hESCs and progenitors in a physiological context [27]. Therefore, it is conceivable that a defective AS machinery may contribute to the acquisition of tumor plasticity, increasing adaptive behavior of cancer cells.

Interestingly, genetic variants affecting the RNA maturation process are as abundant as those affecting gene expression, suggesting the equal contribution of both mutations to phenotypic diversity [51]. In particular, tumor profiling based on analysis of AS events identified tumor-specific splicing patterns.

Taken together, these findings strongly suggest a significant contribution of AS to cancer cell plasticity. We reported several examples showing the intricate connection between AS and activation of EMT, an important process for the acquisition of both invasive capabilities and stemness traits. This evidence explains how defective AS could be a trigger for tumor relapse and metastatic spread, dramatically affecting patient outcome.

Importantly, this review also highlights the involvement of AS events in establishing new mechanisms for glucose uptake during tumor progression. In particular, we showed that an imbalance in isoforms of glycolytic enzymes severely influences the cellular response to metabolic stress. In addition, AS mechanisms significantly affect the balance between pro- and anti-angiogenic factors in the tumor microenvironment, further supporting survival of cancer cells in a challenging microenvironment. 

In summary, AS emerges as an important determinant of cellular plasticity and invasive behavior of cancer cells, which significantly determines a worse outcome in cancer patients. Together these findings strongly support the idea that defective AS mechanisms could have a detrimental role in cancer progression, tumor relapse, and metastasis.

The identification of driver pathways that regulate multiple downstream effectors of common splicing patterns may provide new prognostic and predictive markers for tumor treatment. In addition, it could lead to the development of novel strategies to treat cancer patients, specifically targeting tumor plasticity that is considered the main cause of cancer progression, tumor relapse, and metastatic spread.
